# Mesoscale atmospheric transport of ragweed pollen allergens from infected to uninfected areas

**DOI:** 10.1007/s00484-016-1139-6

**Published:** 2016-02-02

**Authors:** Ł. Grewling, P. Bogawski, D. Jenerowicz, M. Czarnecka-Operacz, B. Šikoparija, C. A. Skjøth, M. Smith

**Affiliations:** 1 Laboratory of Aeropalynology, Faculty of Biology, Adam Mickiewicz University, Umultowska 89, 61-614 Poznań, Poland; 2 Department of Climatology, Faculty of Geographical and Geological Sciences, Adam Mickiewicz University, Dzięgielowa 27, 61-680 Poznań, Poland; 3 Department of Dermatology, University of Medical Science, Przybyszewskiego 49, 60-355 Poznań, Poland; 4 Laboratory for Palynology, Department of Biology and Ecology, Faculty of Sciences, University of Novi Sad, Trg Dositeja Obradovica 2, 21000 Novi Sad, Serbia; 5 BioSense Institute - Institute for Research and Development of Information Technology in Biosystems, Novi Sad, UK; 6 National Pollen and Aerobiological Research Unit, Institute of Science and the Environment, University of Worcester, Henwick Grove, WR2 6AJ Worcester, UK; 7 Institute of Science and the Environment, University of Worcester, Henwick Grove, WR2 6AJ Worcester, UK

**Keywords:** Airborne allergens, Amb a 1, *Ambrosia*, Pollen allergy, Subpollen sized respirable particles

## Abstract

Allergenic ragweed (*Ambrosia* spp.) pollen grains, after being released from anthers, can be dispersed by air masses far from their source. However, the action of air temperature, humidity and solar radiation on pollen grains in the atmosphere could impact on the ability of long distance transported (LDT) pollen to maintain allergenic potency. Here, we report that the major allergen of *Ambrosia artemisiifolia* pollen (Amb a 1) collected in ambient air during episodes of LDT still have immunoreactive properties. The amount of Amb a 1 found in LDT ragweed pollen grains was not constant and varied between episodes. In addition to allergens in pollen sized particles, we detected reactive Amb a 1 in subpollen sized respirable particles. These findings suggest that ragweed pollen grains have the potential to cause allergic reactions, not only in the heavily infested areas but, due to LDT episodes, also in the regions unaffected by ragweed populations.

**Electronic supplementary material:**

The online version of this article (doi:10.1007/s00484-016-1139-6) contains supplementary material, which is available to authorized users.

## Introduction

Four species of ragweed (from the genus *Ambrosia*) have been introduced to Europe from North America. The foremost of these is common ragweed (*Ambrosia artemisiifolia L.*), which is an important weed in agriculture and a source of highly allergenic pollen. It poses a threat to human health, not only in its native North America but also in many parts of the world where it has been introduced. The plant has now become naturalised in Europe, where the largest sources of ragweed pollen are considered to be the Rhône Valley (France), parts of northern Italy, the Pannonian Plain and Ukraine (Smith et al. 2013).

An increase in the distribution (northern range shift) of *A. artemisiifolia* has been observed during recent decades. *A. artemisiifolia* has gradually colonised western Europe (e.g. Germany, Denmark) (Buters et al. 2015; Sommer et al. 2015) as well as the Baltic States (e.g. Lithuania, Latvia and Estonia) (Saar et al. 2000). The plant has been recorded in southern Poland but central Poland is still considered to be ragweed-free i.e. it does not currently have extensive local ragweed populations (Tokarska-Guzik et al. 2011). Historical records show that only one solitary stand of *A. artemisiifolia* has previously been recorded in Poznań (western Poland), but a later survey found that this area was no longer populated (Stach et al. 2007). The most recent ragweed plant inventory of the city was performed during the 2009–2010 flowering seasons but no ragweed stands were observed (Grewling, personal observations). The nearest locations of well-established *A. artemisiifolia* populations are therefore likely to be situated approximately 250 km to the south (Silesia region) and west (Berlin area) (Cunze et al. 2013).

The combined analysis of aerobiological data, meteorological data and air mass trajectory calculations has shown that the majority of ragweed pollen grains recorded in Poznań arrived from south-easterly directions, particularly from the Pannonian Plain (Stach et al. 2007; Smith et al. 2008) and Ukraine (Kasprzyk et al. 2011). Air mass trajectory calculations are used to plot the path along which a parcel of air travels during a certain time interval and have been widely used in aerobiological studies to investigate the transport of airborne pollen from source to receptor areas (Cecchi et al. 2006; Belmonte et al. 2008; Šikoparija et al. 2009; Hernández-Ceballos et al. 2011). Ragweed pollen grains are small (∼20 μm) and have the potential for long distance transport (LDT) when conditions are favourable. With respect to LDT from the Pannonian Plain, the combination of orographic foehn winds, the mesoscale phenomena termed the Kosava (a dry gusty wind with high pressure over the Pannonian Plain) and associated hot weather has been identified as a potential mechanism for aiding ragweed pollen release in the Pannonian Plain and for forcing airborne pollen northward to Poland (Šikoparija et al. 2013).

The allergenic capacity of LDT ragweed pollen remains unclear. Pollen allergenicity could decrease or be lost altogether during flight in the higher layers of the atmosphere, where the action of factors such as air temperature, humidity and solar radiation on the pollen grains could impact on their ability to maintain allergenic potency (Cecchi et al. 2010). The aim of this study is therefore to investigate whether reactive *A. artemisiifolia* pollen allergens (Amb a 1) could be present in ambient air collected during LDT episodes of ragweed pollen.

## Materials and methods

### Monitoring area

The collection of airborne ragweed pollen and atmospheric concentrations of Amb a 1 was performed in Poznań (52.47 N 16.92 E) between the 11 August and the 28 September 2011, which is within the main flowering period of ragweed in Europe. Poznań is the largest city of western Poland with a population of ∼550,000 people (CSO 2013). It is a capital of Wielkopolska, an agricultural region located in mid-western Poland. Poznań has a temperate continental climate with cold winters and warm summers, and westerly winds predominate, particularly from the SW (Woś 2010).

### Collection and quantification of ragweed pollen

*Ambrosia* pollen grains in ambient air (Table S1) were collected by 7-day volumetric trap of Hirst design (Hirst 1952) sited at roof level (18 m a.g.l). Pollen grains were impacted on adhesive transparent tape coated with a mixture of Vaseline, liquid paraffin and toluene and supported on a clockwork-driven drum. The drum moves past an orifice at 2 mm per hour. Deposited pollen grains were mounted on a glass microscope slide, stained with basic fuchsin and covered with a glass cover-slip. Slides were examined by light microscopy (magnification ×400), along four longitudinal transects that were divided into 2 mm (1-hourly) intervals. The sampling time was 12:00–12:00 h in order to be concurrent with allergen sampling times but is described as a “daily average” throughout. Daily average and bi-hourly airborne ragweed pollen concentrations are expressed as particles/m^3^ (P m^−3^) (Mandrioli et al. 1998).

### Collection and quantification of Amb a 1

This study focused on Amb a 1 because this protein is the major allergen of *A. artemisiifolia*; around 90 % of ragweed-sensitive subjects have Amb a 1 specific antibodies (Mohapatra et al. 2008). Atmospheric Amb a 1 was collected using two-stage (stage 1 PM > 10 μm, and stage 2 2.5 > PM > 10 μm) Chemvol® cascade impactor (Butraco Inc., Son, Netherlands) supplied with high-volume (800 l/min) vacuum pump (Digital High Volume air pump DHM60, Ludesch, Austria). The two-stages of the Chemvol® allowed the separation of pollen grain sized particles (stage 1 PM > 10 μm) from the respirable fraction of subpollen sized particles (SSP) (stage 2 2.5 > PM > 10 μm). SSP can easily penetrate into the lower respiratory tract and, due to both antigenic and redox properties, may play an important role in seasonal asthma (Bacsi et al. 2006; Pazmandi et al. 2012). Calculation of Amb a 1 in air samples was performed by antibody-based two-site immunoenzymatic assay. Evaluation of these methods for collecting and quantifying airborne pollen allergens has been performed during the European Union funded HIALINE project (www.hialine.com), thereby confirming their suitability for airborne allergen monitoring (Buters et al. 2012). The device was located next to the Hirst type volumetric trap (2 m apart). The polyurethane filters used in the Chemvol® sampler as impacting substrate were prepared according the method described in Buters et al. (2010). The sampling time was 12:00–12:00 h. After 24 h, the filters were removed from the Chemvol® and cut in three equally sized pieces. The airborne material collected on the filters was extracted in a darkened room over a 4-h period using 0.1 M ammonium bicarbonate buffer (NH_4_) HCO_3_ pH 8.1 and lyophilized. Lyophilized material was dissolved in 1/10 of the original volume in phosphate-buffered saline (PBS). Determination of allergen concentrations was performed using enzyme-linked immunosorbent assay (ELISA) following the protocol supplied by Indoor Biotechnologies company (Amb a1 ELISA kit, EL-AM1), with two exceptions: (i) the streptavidin-peroxidase (Sigma-Aldrich S5512) was used as an enzyme; (ii) the 3,3′5,5′-tetramethylobenzidine (Sigma-Aldrich T0440) was used as a substrate. The reaction was stopped by adding 2.5 M H_2_SO_4_ (5 N). The concentration of the Amb a 1 in the sample was determined by reading the absorbance at 450 nm. All samples were determined in duplicate. Each assay was calibrated with a standard curve and included with two controls. Interassay variability was 14 % (*n* = 14) in the higher range and 13.2 % (*n* = 14) in the lower range of the calibration curve. All extracts were diluted so that the values were within the linear range of the calibration curve. Reported values for each day are the mean of two filter parts. If the coefficient of variance between values from two filter segments were >25 %, the concentration of Amb a 1 in the third filter segment was determined and included into analysis. Daily average concentrations of Amb a 1 are expressed as pg m^−3^.

### Back-trajectory analysis

Back-trajectories were computed using a cluster approach that was first used on aerobiological data by Stach et al. (2007) and subsequently employed for numerous studies concerned with airborne pollen and fungal spores (e.g. Skjøth et al. (2012) and references therein). There is a definite diurnal periodicity in common ragweed flowering, with peak concentrations of ragweed pollen having been reported from approximately 06:30 to around midday in field studies (Smith et al. (2013) and references therein). As a result, investigations into the long distance transport of ragweed pollen are usually based on the premise that ragweed pollen recorded during nighttime is less likely to originate from local sources. The fact that Poznań is located in an area considered to be devoid of large local ragweed populations and situated approximately 250 km from the northern edge of *A. artemisiifolia* distribution (Cunze et al. 2013) supports the assumption that the ragweed pollen recorded during this study originated from distant areas. In addition, the study conducted by Šikoparija et al. (2013) described the northward progress of a plume of ragweed pollen from the Pannonian Plain to Scandinavia on the 27–28 August 2011, which is one of the episodes examined in this study.

Here, we use the Lagrangian Integrated Trajectory (HYSPLIT) model (Draxler et al. 2013) with the GDAS (Global Data Analysis System) meteorological files as input having a temporal resolution of 3 h and a spatial resolution of 1°. Trajectories were calculated at Poznań for all episodes with a receiving height of 500, 1000 and 1500 m to take into account variations in air mass directions with increasing height in the atmosphere. Trajectories were calculated 48 h back in time with 2 h steps between each trajectory (thus 12 trajectories each day), which corresponds to the time step of the bi-hourly pollen observations (Stach et al. 2007).

### Meteorological data

Weather data with hourly resolution were recorded by a Davis Vantage Pro 2 meteorological station to produce maximum, minimum and mean temperatures (°C), relative humidity (%), sum of rainfall (mm), wind speed (m/s) and dew point (°C). The meteorological station was located between the Hirst (pollen) and Chemvol® (Amb a 1) sampling devices. As with the pollen and allergen sampling, meteorological parameters included in the statistical analysis were recorded from midday to midday and described as “daily averages” throughout (Table S2).

### Statistical analysis

In order to obtain more robust results (due to the uncertainties in pollen counts and allergen determination), only days with daily average ragweed pollen levels >3 P m^−3^ and/or Amb a 1 > 20 pg m^−3^ were taken into account in statistical analysis (*n* = 9). Nonparametric Spearman’s rank correlation analysis was used to examine relationships between daily average pollen concentrations, daily average Amb a 1 levels in both air fractions, pollen potency and meteorological parameters. Differences between the level of Amb a 1 in PM > 10 μm and 2.5 > PM > 10 μm air fractions were analysed by the Wilcoxon signed-rank test. The statistical significance of the results was determined by comparing the *p* value with the significance level (α = 0.05). The amount of Amb a 1 in one ragweed pollen grain (so-called pollen potency or the allergenicity of pollen) was calculated by dividing the daily average Amb a 1 concentrations (detected in both air fractions) by the daily average ragweed pollen level. All calculations were carried out with the Statistica 10 software package.

## Results

**Fig. 1:**
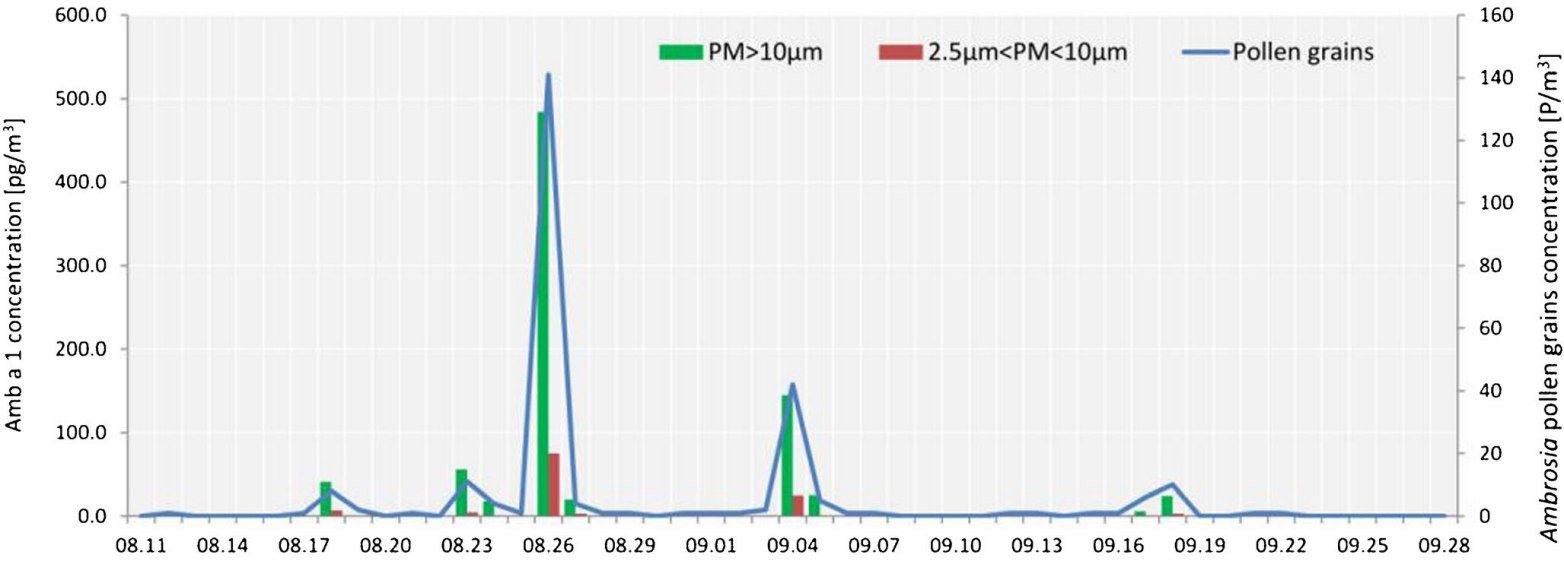
Variations in daily average concentrations of airborne ragweed pollen and the major allergen of *A. artemisiifolia* pollen (Amb a 1) detected in two air fractions (PM > 10 μm and 2.5 μm < PM < 10 μm) in Poznań, 2011

Ragweed pollen grains were collected for 28 days during the sampling period in 2011 (Table S1). Daily average ragweed pollen concentrations were generally low (17 days with 1 P m^−3^) (Fig. 1). However, three distinctly separated peaks in *Ambrosia* pollen concentrations were observed: (I) 23–27 of August; (II) 4–5 September; and (III) 17–18 September (Table 1). We detected reactive Amb a 1 in 32 % of the samples collected on days when ragweed pollen was recorded. Amb a 1 was recorded on every day with daily average ragweed pollen concentration >3 P m^−3^ and only these days were taken into account in the statistical analysis. Daily average Amb a 1 levels varied from 5.9 to 559.4 pg m^−3^ and were significantly correlated with daily average ragweed pollen concentrations (Spearman correlation coefficient = 0.879; *p* = 0.003, Table 2). The level of Amb a 1 detected in SSP was significantly lower (Wilcoxon signed-rank test; *Z* = 2.666, *p* = 0.008) than the Amb a 1 in pollen grain sized particles. On average, seven times more Amb a 1 was found in stage 1 compared to that found in stage 2. The ratio between the level of Amb a 1 in both air fractions (expressed in %) varied between 0 (no Amb a 1 in stage 2) to 17.2 %. The mean seasonal ragweed pollen potency was 4.3 pg Amb a 1/pollen grain, which is similar to the results obtained for the major allergens of birch and olive recorded at sites belonging to the HIALINE network (2.5–3.9 pg Bet v 1/pollen and 0.8–3.9 pg Ole e 1/pollen, respectively) (Buters et al. 2012; Galán et al. 2013). It should be noted that allergenicity of airborne ragweed pollen varied between different days (from 0.98 to 5.98 pg Amb a 1/pollen) as well as between the three different pollen peaks—from 1.86 pg Amb a 1/pollen (episode III) to 4.97 pg Amb a 1/pollen (episode I) (Table 1).

**Table 1:** Cumulative totals of ragweed pollen and ragweed pollen allergens recorded during the whole pollen season and during the three LDT episodes of ragweed pollen in Poznań during 2011 season

Period	*Ambrosia* pollen [P m^−3^]	Amb a 1 in stage 1 [pg m^−3^]	Amb a 1 in stage 2 [pg m^−3^]	Sum of Amb a 1 in both fraction [pg m^−3^]	Pollen potency [pg Amb a 1/pollen]
Episode I (23–27 August)	161	578.8	82.8	661.6	5.0
Episode II (4–5 September)	47	170.1	25.0	195.1	4.5
Episode III (17–18 September)	16	32.1	3.1	35.2	1.9
Season	252	819.9	118.0	937.8	4.3

**Table 2:** Spearman’s rank correlation coefficients between aerobiological and meteorological parameters (*n* = 9)

	Amb a 1 in stage 1 [pg m^−3^]	Amb a 1 in stage 2 [pg m^−3^]	Sum of Amb a 1 [pg m^−3^]	Pollen potency [pg Amb a 1/pollen]	Amb a 1 in stage 2/stage 1 [%]
Daily average *Ambrosia* pollen [P m^−3^]	*0.828*	*0.816*	*0.879*	−0.310	0.579
Amb a 1 in stage 1 [pg m^−3^]	–	*0.877*	*0.983*	0.167	*0.678*
Amb a 1 in stage 2 [pg m^−3^]	*0.877*	–	*0.919*	0.136	*0.892*
Sum of Amb a 1 [pg m^−3^]	*0.983*	*0.919*	–	0.100	*0.729*
Pollen potency [pg Amb a 1/pollen]	0.167	0.136	0.100	–	0.237
Amb a 1 in stage 1/stage 2 [%]	*0.678*	*0.892*	*0.729*	0.237	–
Mean daily temperature (°C)	0.600	0.638	0.567	0.117	0.475
Maximum daily temperature (°C)	0.550	0.613	0.483	0.483	0.627
Minimum daily temperature (°C)	0.444	0.410	0.427	0.000	0.119
Daily relative humidity (%)	−0.533	−0.570	−0.500	−0.250	−0.542
Daily average dew point (°C)	0.417	0.255	0.333	0.267	0.153
Daily average wind speed (m/s)	0.500	0.460	0.450	−0.200	0.390
Daily sum of rainfall (mm)	−0.366	−0.578	−0.400	0.252	−0.478

Statistically significant correlations (*p* < 0.05) are in italics

**Fig. 2:**
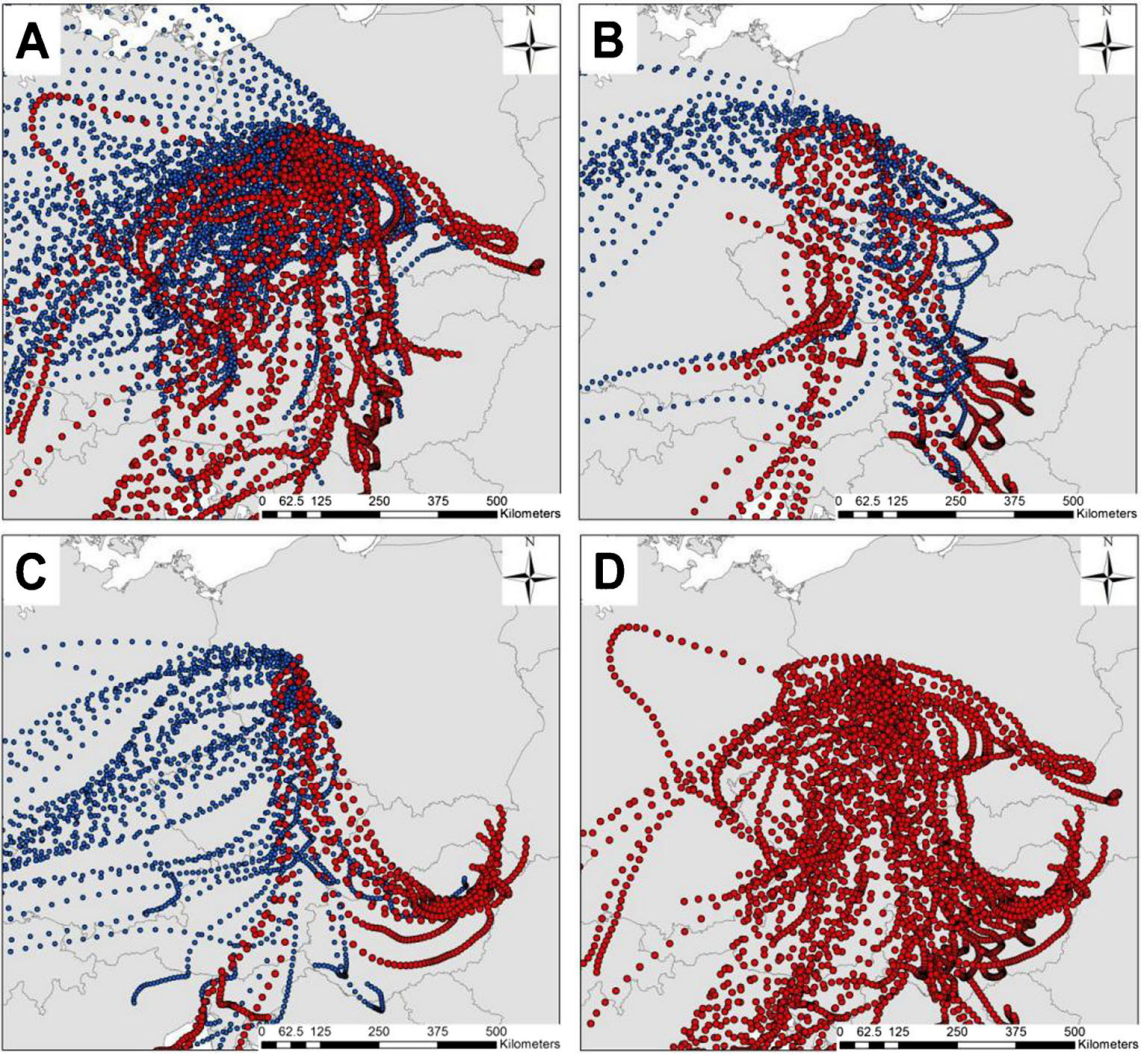
Back-trajectories calculated for the LDT episodes investigated in Poznań during 2011. Trajectories were calculated for 48 h back in time with 2 h steps for the following 24 h periods: **a** Episode I 23–27 of August. **b** Episode II 4–5 September. **c** Episode III 17–18 September. **d** All trajectories calculation during the investigated episodes (I–III) when bi-hourly ragweed pollen concentrations exceeded 5 P m^−3^ (See also Table S1). Note that trajectories bringing ragweed pollen (*red*) generally arrived from the south, particularly in the direction of the Pannonian Plain. Whereas trajectories calculated when no, or very little (<5 P m^−3^), ragweed pollen arrived in Poznań (*blue* in Figs **a**, **b** and **c**) tended to approach the city from areas that are not considered to be centres of ragweed infestation (e.g. Germany to the west). Trajectories plotted for individual days are shown in the Supplementary Information (Fig. S1)

In order to determine whether the observed differences in ragweed pollen potency were the result of the ragweed pollen originating from different source areas, we have examined air mass trajectories based on meteorological calculations from the GDAS reanalyzed meteorological dataset in combination with the HYSPLIT trajectory model (Draxler and Hess 1998). Back-trajectory calculations (Fig. 2a–d) show that air masses that brought ragweed pollen to Poznań (bi-hourly ragweed pollen concentrations >5 P m^−3^), usually arrived from a southerly direction after passing over southern Poland, Czech Republic, central and eastern Austria and Hungary. On the other hand, the amount of ragweed pollen recorded in Poznań dropped to very low levels (bi-hourly ragweed pollen concentrations ≤5 P m^−3^) when air masses approached from areas not considered to be centres of ragweed infestation like Germany to the west.

## Discussion

The results of this study reveal large day-to-day variations in ragweed pollen potency. Similar variability has also been observed in relation to birch (Buters et al. 2010, 2012) and olive (Galán et al. 2013). As shown for olive, variations in the allergenicity of pollen grains may depend on the source area (Galán et al. 2013), which is possibly related to the allergen content of different olive cultivars (Ribeiro et al. 2013). There is lack of data about differences in the allergenicity of pollen from different ragweed populations, but nuclear and chloroplast microsatellite data (Gaudeul et al. 2011) suggest that European ragweed populations originating from different regions of North America show high levels of diversity. Back-trajectory analysis showed that air masses arriving in Poznań during the three LDT episodes examined, generally came from the south and originated in central Europe, particularly the Pannonian Plain. As a result, observed differences in ragweed pollen potency cannot be linked with differences in the allergenicity of ragweed pollen originating from different source areas. In addition, variations in ragweed pollen potency did not show statistically significant relationships with weather conditions recorded during LDT episodes (Table 2). The cause of these observed differences in the allergenicity of ragweed pollen therefore remains unclear.

The major allergen of *A. artemisiifolia* pollen was also detected in the SSP samples, which concurs with previous findings (Busse et al. 1972; Agarwal et al. 1983; Solomon et al. 1983; Habenicht et al. 1984). There is a paucity of data concerning the mechanism of Amb a 1 release from pollen grains (Oswalt and Marshall 2008). Although increases in the amount of airborne ragweed pollen allergens found in the SSP fraction have been linked with higher atmospheric water vapour (Habenicht et al. 1984). Similar results were seen for birch and grass pollen allergens (Schäppi et al. 1997, 1999). Amb a 1 (pectate lyase) is synthesised and released when the pollen tube grows, and so moist conditions at any point of pollen flight (from anthesis to capture on the Chemvol® filters) would facilitate this process (Dearnaley and Daggard 2001). We did not find such a relationship between SSP and atmospheric moisture content (Table 2), but this might be because LDT episodes are often associated with hot and dry weather (Table S2) (Šikoparija et al. 2013). Previous work has also shown that ragweed pollen allergens can be detected in the air when no ragweed pollen is present (Agarwal et al. 1984). Our work does not support these earlier findings. One possible reason for this, is the fact that the dry weather experienced during the atmospheric transport of ragweed pollen from central to northern Europe might delay the release of allergens until the pollen are collected in the sampler.

It is projected that climate change will facilitate an expansion and northwards shift in the potential range of ragweed in central and northern Europe during the next decades (Cunze et al. 2013; Leiblein-Wild et al. 2014). In addition, it is suspected that, due to higher temperatures and CO_2_ levels, ragweed pollen seasons will become longer (Ziska et al. 2011) and pollen production as well as allergenicity will increase (Ziska and Caulfield 2000; Singer et al. 2005; Rogers et al. 2006). Sensitization to ragweed pollen has increased in areas infested by ragweed populations and coincides with ragweed pollen seasons becoming more intense (Jäger 2000; Albertini et al. 2012). It is important to note that, due to LDT episodes, it is not only areas that are heavily infested with ragweed that experience high ragweed pollen concentrations (Prank et al. 2013). For the first time, our study has shown that LDT ragweed pollen still contains reactive Amb a 1. These pollen grains are therefore equipped to induce allergic reactions in sensitised patients and, if the frequency and magnitude of the episodes are sufficient, LDT ragweed pollen has the potential to induce new sensitizations in areas currently unaffected by ragweed populations. Ragweed pollen has the potential to be transported hundreds of kilometres. Areas at risk from the centres of ragweed distribution in Europe include much of the European Continent as far as Turkey to the south (Zemmer et al. 2012), Spain to the west (Belmonte et al. 2000), and even Scandinavia to the north (e.g. Dahl et al. 1999, Šikoparija et al. 2013; Sommer et al. 2015). This is also relevant for other parts of the world, such as North America, eastern Asia and Australia where ragweed is considered to be an environmental health problem (Bass et al. 2000; Xie et al. 2004; Ziska et al. 2011).

## Conclusions

Ragweed plants produce highly allergenic pollen that, after being released from flowers, can be transported by air masses far from their source. We show that these pollen grains still possess reactive allergens, even after spending days in the atmosphere. These findings suggest that exposed individuals may become sensitised to ragweed pollen allergens and could develop symptoms, even in areas where the plant is not widely distributed. This also has an impact on pollen allergy sufferers that try to avoid exposure to ragweed pollen allergens by moving to areas some distance from the source.

## Electronic supplementary material

Below is the link to the electronic supplementary material.

ESM 1 (DOCX 1439 kb)
