# Supplementary material for: Mesoscale atmospheric transport of ragweed pollen allergens from infected to uninfected areas
Source: Int J Biometeorol. 2016 Feb 2;60(10):1493–500. doi: 10.1007/s00484-016-1139-6 (PMC5050238; doi:10.1007/s00484-016-1139-6)
Supplement: Supplementary file 1 — (DOCX 1439 kb) [file 484_2016_1139_MOESM1_ESM.docx]

Title: Mesoscale atmospheric transport of ragweed pollen allergens from infected to uninfected areas

**Authors:** Ł. Grewling^1*^, P. Bogawski^1^, D. Jenerowicz^2^, M. Czarnecka-Operacz^2^, B. Šikoparija^3^, C. A. Skjøth^4^, M. Smith^1^

**Affiliations:**

^1^Laboratory of Aeropalynology, Faculty of Biology, Adam Mickiewicz University, Umultowska 89, 61-614 Poznań, Poland.

^2^Department of Dermatology, 60-355 Poznań, Przybyszewskiego 49, University of Medical Science, Poznań, Poland.

^3^Laboratory for Palynology, Department of Biology and Ecology, Faculty of Sciences, University of Novi Sad, Trg Dositeja Obradovica 2, 21000 Novi Sad, Serbia.

^4^National Pollen and Aerobiological Research Unit, Institute of Science and the Environment University of Worcester, Henwick Grove, WR2 6AJ, Worcester, UK.

*Correspondence to: Łukasz Grewling, Adam Mickiewicz University, Faculty of Biology, Laboratory of Aeropalynology, Umultowska 89, 61-614 Poznań, Poland, e-mail: [lukaszgrewling@gmail.com](mailto:lukaszgrewling@gmail.com)

Key words: airborne allergens, Amb a 1, *Ambrosia*, pollen allergy, subpollen sized respirable particles

**Supplementary Information**

Fig. S1. Back-trajectories calculated for the LDT episodes investigated in Poznań during 2011. Trajectories were calculated for 48 h back in time with 2 h steps for the following 24 hour periods: (A) Episode I - 23-27 of August; (B) Episode II - 4-5 September; (C) Episode III -17-18 September.


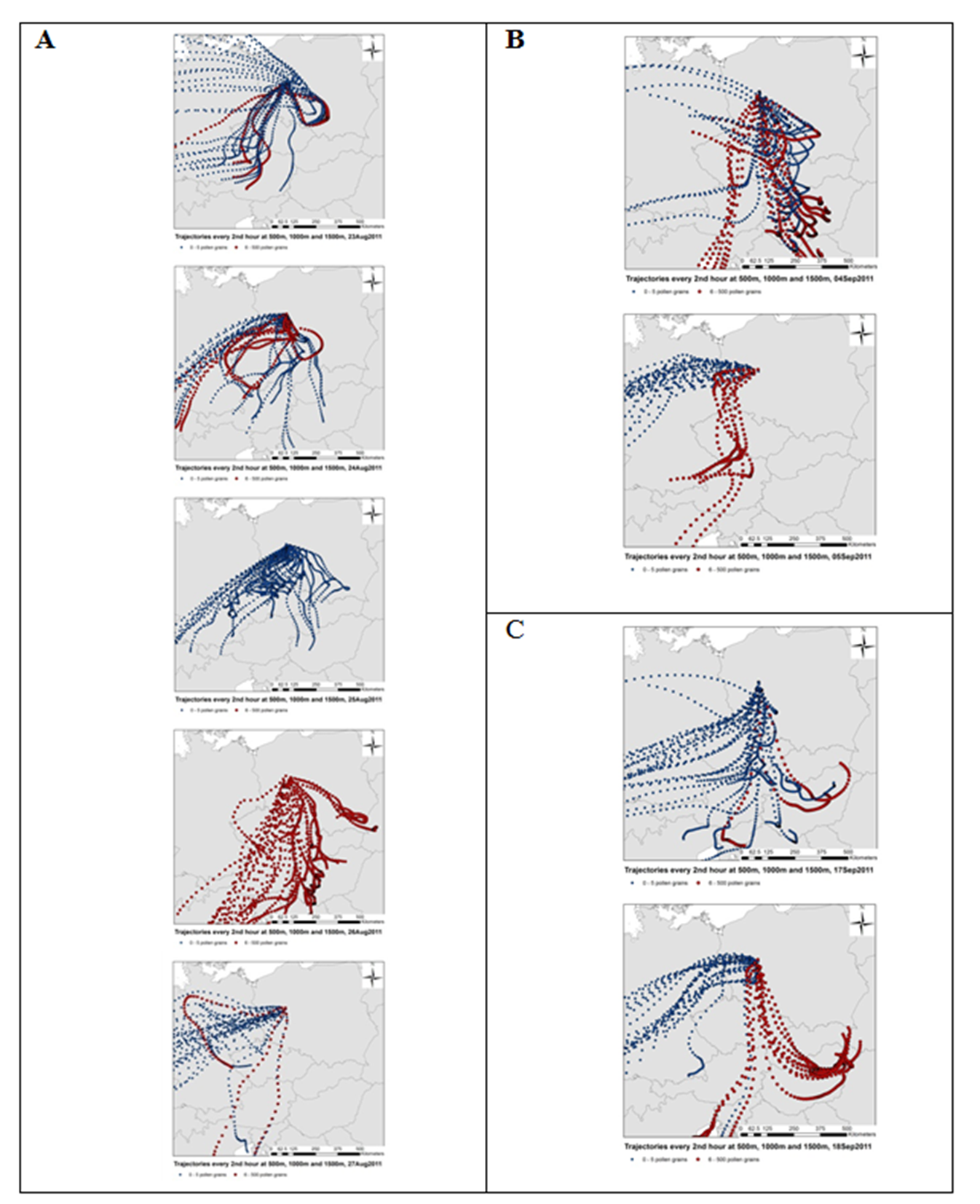


**Table S1**. Bi-hourly ragweed pollen concentrations recorded in Poznań, 2011. Those marked with bold (red or blue) corresponds to the trajectories (red or blue) in Fig 2. Note that the time period (midday to midday) corresponds with the allergen sampling. The episodes investigated are shaded light grey. DA = Daily Average

| DATE | 12:00 - 14:00 | 14:00-16:00 | 16:00-18:00 | 18:00-20:00 | 20:00-22:00 | 22:00-24:00 | 00:00 -02:00 | 02:00- 04:00 | 04:00- 06:00 | 06:00- 08:00 | 08:00 - 10:00 | 10:00-12:00 | DA |
| --- | --- | --- | --- | --- | --- | --- | --- | --- | --- | --- | --- | --- | --- |
| 2011-08-11 | 0 | 0 | 0 | 0 | 0 | 0 | 0 | 0 | 0 | 0 | 0 | 0 | **0** |
| 2011-08-12 | 0 | 0 | 0 | 0 | 0 | 0 | 0 | 0 | 11 | 0 | 0 | 0 | **1** |
| 2011-08-13 | 0 | 0 | 0 | 0 | 0 | 0 | 0 | 0 | 0 | 0 | 0 | 0 | **0** |
| 2011-08-14 | 0 | 0 | 0 | 0 | 0 | 0 | 0 | 0 | 0 | 0 | 0 | 0 | **0** |
| 2011-08-15 | 0 | 0 | 0 | 0 | 0 | 0 | 0 | 0 | 0 | 0 | 0 | 0 | **0** |
| 2011-08-16 | 0 | 0 | 0 | 0 | 0 | 0 | 0 | 0 | 0 | 0 | 0 | 0 | **0** |
| 2011-08-17 | 0 | 0 | 0 | 0 | 0 | 0 | 0 | 0 | 0 | 0 | 5 | 0 | **1** |
| 2011-08-18 | 0 | 0 | 0 | 0 | 0 | 0 | 0 | 0 | 21 | 74 | 5 | 0 | **8** |
| 2011-08-19 | 0 | 11 | 11 | 0 | 0 | 0 | 0 | 0 | 0 | 0 | 0 | 0 | **2** |
| 2011-08-20 | 0 | 0 | 0 | 0 | 0 | 0 | 0 | 0 | 0 | 0 | 0 | 0 | **0** |
| 2011-08-21 | 0 | 5 | 0 | 0 | 0 | 0 | 0 | 0 | 0 | 0 | 0 | 0 | **1** |
| 2011-08-22 | 0 | 0 | 0 | 0 | 0 | 0 | 0 | 0 | 0 | 0 | 0 | 0 | **0** |
| 2011-08-23 | **0** | **0** | **0** | **0** | **0** | **0** | **0** | **0** | **0** | **11** | **48** | **80** | **11** |
| 2011-08-24 | **0** | **5** | **0** | **0** | **0** | **0** | **27** | **11** | **5** | **0** | **0** | **0** | **4** |
| 2011-08-25 | **5** | **0** | **0** | **5** | **0** | **0** | **0** | **0** | **5** | **0** | **0** | **0** | **1** |
| 2011-08-26 | 37 | 37 | 37 | 58 | 64 | 53 | **101** | **313** | **482** | **339** | **154** | **27** | **141** |
| 2011-08-27 | **37** | **5** | **5** | **0** | **0** | **0** | 0 | 0 | 0 | 5 | 0 | 0 | **4** |
| 2011-08-28 | 0 | 0 | 0 | 0 | 0 | 5 | 0 | 0 | 0 | 0 | 0 | 0 | **1** |
| 2011-08-29 | 0 | 0 | 0 | 0 | 0 | 0 | 0 | 0 | 0 | 0 | 0 | 5 | **1** |
| 2011-08-30 | 0 | 0 | 0 | 0 | 0 | 0 | 0 | 0 | 0 | 0 | 0 | 0 | **0** |
| 2011-08-31 | 0 | 0 | 0 | 0 | 0 | 0 | 5 | 0 | 0 | 0 | 0 | 0 | **1** |
| 2011-09-01 | 5 | 0 | 0 | 0 | 0 | 0 | 0 | 0 | 0 | 0 | 0 | 5 | **1** |
| 2011-09-02 | 0 | 0 | 0 | 0 | 0 | 0 | 0 | 0 | 0 | 5 | 0 | 0 | **1** |
| 2011-09-03 | 0 | 0 | 0 | 0 | 0 | 0 | 0 | 5 | 0 | 11 | 5 | 0 | **2** |
| 2011-09-04 | **0** | **16** | **0** | **5** | **0** | **0** | **5** | **16** | **85** | **159** | **106** | **117** | **42** |
| 2011-09-05 | **42** | **21** | **0** | **0** | **0** | **0** | **0** | **0** | **0** | **0** | **0** | **0** | **5** |
| 2011-09-06 | 0 | 0 | 0 | 0 | 0 | 0 | 11 | 0 | 0 | 0 | 0 | 0 | **1** |
| 2011-09-07 | 5 | 0 | 0 | 0 | 0 | 0 | 0 | 0 | 0 | 0 | 0 | 0 | **1** |
| 2011-09-08 | 0 | 0 | 0 | 0 | 0 | 0 | 0 | 0 | 0 | 0 | 0 | 0 | **0** |
| 2011-09-09 | 0 | 0 | 0 | 0 | 0 | 0 | 0 | 0 | 0 | 0 | 0 | 0 | **0** |
| 2011-09-10 | 0 | 0 | 0 | 0 | 0 | 0 | 0 | 0 | 0 | 0 | 0 | 0 | **0** |
| 2011-09-11 | 0 | 0 | 0 | 0 | 0 | 0 | 0 | 0 | 0 | 0 | 0 | 0 | **0** |
| 2011-09-12 | 0 | 0 | 0 | 0 | 0 | 0 | 0 | 0 | 0 | 0 | 5 | 0 | **1** |
| 2011-09-13 | 0 | 0 | 5 | 0 | 0 | 0 | 0 | 0 | 0 | 0 | 0 | 0 | **1** |
| 2011-09-14 | 0 | 0 | 0 | 0 | 0 | 0 | 0 | 0 | 0 | 0 | 0 | 0 | **0** |
| 2011-09-15 | 5 | 0 | 0 | 0 | 0 | 0 | 0 | 0 | 0 | 0 | 0 | 0 | **1** |
| 2011-09-16 | 0 | 0 | 0 | 0 | 0 | 0 | 0 | 0 | 0 | 0 | 5 | 0 | **1** |
| 2011-09-17 | **5** | **0** | **0** | **0** | **0** | **0** | **0** | **0** | **0** | **0** | **0** | **69** | **6** |
| 2011-09-18 | **53** | **27** | **21** | **16** | **5** | **0** | **0** | **0** | **0** | **0** | **0** | **0** | **10** |
| 2011-09-19 | 0 | 0 | 0 | 0 | 0 | 0 | 0 | 0 | 0 | 0 | 0 | 0 | **0** |
| 2011-09-20 | 0 | 0 | 0 | 0 | 0 | 0 | 0 | 0 | 0 | 0 | 0 | 0 | **0** |
| 2011-09-21 | 0 | 0 | 0 | 5 | 0 | 5 | 5 | 0 | 0 | 0 | 0 | 0 | **1** |
| 2011-09-22 | 5 | 0 | 0 | 0 | 0 | 0 | 0 | 0 | 0 | 0 | 0 | 0 | **1** |
| 2011-09-23 | 0 | 0 | 0 | 0 | 0 | 0 | 0 | 0 | 0 | 0 | 0 | 0 | **0** |
| 2011-09-24 | 0 | 0 | 0 | 0 | 0 | 0 | 0 | 0 | 0 | 0 | 0 | 0 | **0** |
| 2011-09-25 | 0 | 0 | 0 | 0 | 0 | 0 | 0 | 0 | 0 | 0 | 0 | 0 | **0** |
| 2011-09-26 | 0 | 0 | 0 | 0 | 0 | 0 | 0 | 0 | 0 | 0 | 0 | 0 | **0** |
| 2011-09-27 | 0 | 0 | 0 | 0 | 0 | 0 | 0 | 0 | 0 | 0 | 0 | 0 | **0** |
| 2011-09-28 | 0 | 0 | 0 | 0 | 0 | 0 | 0 | 0 | 0 | 0 | 0 | 0 | **0** |

**Table S2.** Meteorological parameters recorded in Poznań during LDT episodes of atmospheric ragweed pollen when daily average concentrations exceeded 3Pm^-3^.

| Date | Temp. mean (°C) | Temp. max. (°C) | Temp. min. (°C) | Relative humidity (%) | Dew point (°C) | Wind speed (m/s) | Sum of rain (mm) |
| --- | --- | --- | --- | --- | --- | --- | --- |
| 08.18 | 23.1 | 28.8 | 18.1 | 58.2 | 14.0 | 1.5 | 0.0 |
| 08.23 | 20.6 | 23.9 | 18.1 | 61.5 | 12.7 | 2.0 | 0.2 |
| 08.24 | 21.3 | 26.6 | 18.5 | 85.2 | 18.6 | 1.1 | 1.4 |
| 08.26 | 26.4 | 29.9 | 22.9 | 71.1 | 20.6 | 3.4 | 0.0 |
| 08.27 | 16.5 | 28.6 | 11.6 | 83.5 | 13.5 | 3.1 | 4.8 |
| 09.04 | 21.9 | 27.5 | 17.2 | 60.5 | 13.6 | 3.3 | 0.0 |
| 09.05 | 17.4 | 27.1 | 12.2 | 82.4 | 14.2 | 2.5 | 6.6 |
| 09.17 | 18.2 | 22.1 | 14.8 | 70.5 | 12.5 | 2.4 | 0.0 |
| 09.18 | 15.6 | 22.0 | 11.7 | 83.7 | 12.7 | 2.0 | 3.4 |
